# A Genome-Wide Association Study of Anti-Müllerian Hormone (AMH) Levels in Samoan Women

**DOI:** 10.3390/genes16070793

**Published:** 2025-06-30

**Authors:** Zeynep Erdogan-Yildirim, Jenna C. Carlson, Mohanraj Krishnan, Jerry Z. Zhang, Geralyn Lambert-Messerlian, Take Naseri, Satupaitea Viali, Nicola L. Hawley, Stephen T. McGarvey, Daniel E. Weeks, Ryan L. Minster

**Affiliations:** 1 Center for Craniofacial and Dental Genetics, Department of Oral and Craniofacial Sciences, School of Dental Medicine, University of Pittsburgh, Pittsburgh, PA 15261, USA; 2 Department of Human Genetics, School of Public Health, University of Pittsburgh, Pittsburgh, PA 15261, USA; 3 Department of Biostatistics and Health Data Science, School of Public Health, University of Pittsburgh, Pittsburgh, PA 15261, USA; 4 Pathology and Laboratory Medicine, Department of Obstetrics and Gynecology, Brown University, Providence, RI 02912, USA; 5 Naseri & Associates Public Health Consultancy Firm and Family Health Clinic, Matafele WS1339, Samoa; 6 International Health Institute and Department of Epidemiology, School of Public Health, Brown University, Providence, RI 02903, USA; 7 Faculty of Medicine, Oceania University of Medicine, Samoa Campus, Apia P.O. Box 232, Samoa; 8 School of Medicine, National University of Samoa, Apia P.O. Box 1622, Samoa; 9 Department of Chronic Disease Epidemiology, School of Public Health, Yale University, New Haven, CT 06520, USA

**Keywords:** anti-Müllerian hormone, AMH, genome-wide association study, reproductive health, Pacific Island People

## Abstract

**Background/Objectives**: The anti-Müllerian hormone (AMH) is a key biomarker of the ovarian reserve, correlating with ovarian follicle count, fertility outcomes, and menopause timing. Understanding its genetic determinants has broad implications for female reproductive health. However, prior genome-wide association studies (GWASs) have focused exclusively on women of European ancestry, limiting insights into diverse populations. **Methods**: We conducted a GWAS to identify genetic loci associated with circulating AMH levels in a sample of 1185 Samoan women from two independently recruited samples. Using a Cox mixed-effects model we accounted for AMH levels below detectable limits and meta-analysed the summary statistics using a fixed-effect model. To prioritize variants and genes, we used FUMA and performed colocalization and transcriptome-wide association analysis (TWAS). We also assessed whether any previously reported loci were replicated in our GWAS. **Results**: We identified eleven genome-wide suggestive loci, with the strongest signal at *ARID3A* (19-946163-G-C; *p* = 2.32 × 10^−7^) and replicated rs10093345 near *EIF4EBP1.* The gene-based testing revealed *ARID3A* and *R3HDM4* as significant genes. Integrating GWAS results with expression quantitative trait loci via TWAS, we detected seven transcriptome-wide significant genes. The lead variant in *ARID3A* is in high linkage disequilibrium (*r*^2^ = 0.79) with the known age-at-menopause variant 19-950694-G-A. Nearby *KISS1R* is a biologically plausible candidate gene that encodes the kisspeptin receptor, a regulator of ovarian follicle development linked to AMH levels. **Conclusions**: This study expands our understandings of AMH genetics by focusing on Samoan women. While these findings may be particularly relevant to Pacific Islanders, they hold broader implications for reproductive phenotypes such as the ovarian reserve, menopause timing, and polycystic ovary syndrome.

## 1. Introduction

Anti-Müllerian hormone (AMH) has an important role in ovarian biology and female reproductive health. AMH is produced in women after birth and is exclusively secreted by the granulosa cells of developing ovarian follicles until the antral stage is reached [1,2,3]. In recent years, researchers have focused on the clinical application of AMH as a surrogate marker to evaluate polycystic ovaries and to diagnose polycystic ovary syndrome (PCOS), a common disorder affecting fertility and metabolic health of women [4,5,6]. Studies have consistently shown that AMH levels are higher in all PCOS subtypes compared to normo-ovulatory women and women with polycystic ovarian morphology alone [7] and that AMH levels correlate with PCOS subtype and severity [8,9]. Hence, AMH could provide a non-invasive alternative to transvaginal ultrasound for antral follicle counts, particularly when the latter is not available or not feasible due to cost and or acceptability (i.e., due to cultural and psycho-social reasons, especially for adolescents) [10].

Our research group has been focused on the health of Samoans, a founder population, for more than thirty years. We have conducted several epidemiological studies to describe the influences of adiposity on Samoan women’s reproductive health, specifically describing menstrual irregularity, hyperandrogenemia, and estimating prevalence of PCOS [11,12,13]. This is especially important given the high and rising levels of adiposity among Samoan women that are characteristic of Pacific Islanders more broadly [14,15,16]. Recently, two distinct PCOS subtypes (metabolic vs. reproductive) with distinct genetic architecture have been described in individuals of European ancestry [17], and it was found that PCOS susceptibility loci differ between lean and overweight/obese cases [18]. We do not have diagnoses of PCOS in Samoan women, and so we cannot directly examine the genetic determinants of PCOS in this study. However, it could be fruitful to examine related phenotypes to begin to understand genetic determinants of reproductive health in this population.

There have been several studies of the genetic variation underlying AMH levels [19,20,21]. In addition to polymorphisms within the *AMH* gene itself [21,22,23], GWASs have mapped seven genes in women of European ancestry, of which four are implicated in cell cycle regulation (*MCM8* [20,21,23], *TEX41* [21,23], *CHECK2* [23], and *CDCA7* [21]). The other three genes are *EIF4EBP1* [23], *BMP4* [23], and an uncharacterized non-coding RNA gene (*CTB-99A3.1* [21]). The established loci explain about 13% to 15% of the single nucleotide variant (SNV)-based heritability [21,23]. A major limitation of the existing studies, however, is that the information on the genetic underpinnings of AMH have been derived solely from women of European ancestry [24,25]. More research is needed in diverse populations not only to enhance our understanding of the underlying biology but also to ensure access to adequate health care and effective treatment for these communities. Importantly, the inclusion of founder populations in genetic research is important since the reduced allelic heterogeneity in these groups can be advantageous to discover novel loci via genome-wide association studies [14,15,16,26,27].

In this study, we aimed to identify genetic determinants of circulating AMH levels via genome-wide and transcriptome-wide analyses in Samoan women. 

## 2. Materials and Methods

### 2.1. Study Subjects

Two independent study samples comprised a total of 1185 Samoan women were selected for a GWAS to assess the genetic variation associated with circulating serum AMH levels. The first sample of 212 women aged ≥ 18 years and < 40 years was drawn from a 2002–2003 family study of genetic linkage analysis of cardiometabolic traits (for sample flowchart see Supplementary Figure S1) [13,28,29]. The age range was limited to reproductive-aged women in the parent study [13] to avoid potential effects of perimenopause. Participants were recruited from villages across ‘Upolu and Savai‘i, the two largest islands of Samoa, and Tutuila, the largest island of American Samoa.

The second sample of 973 Samoan women aged ≥ 25 to ≤50 years was drawn from a 2010 cross-sectional population-based study (Soifua Manuia [in Samoan: “Good Health”] Study) of obesity and cardiometabolic health [12,15,30] (for sample see Supplementary Figure S1). Participants were recruited from thirty-three villages across ‘Upolu and Savai‘i. All participants completed a questionnaire surveying their health history and lifestyle factors related to cardiometabolic and reproductive health including socio-economic status, dietary intake, and physical activity [13,28,29].

In both studies, women who had a history of hysterectomy and/or ovariectomy or who were pregnant or lactating at the time of recruitment were excluded. Hormonal contraceptive use was not well captured in our cohorts for use as an exclusion criterion. The baseline characteristics between individuals with measured and unmeasured AMH levels are compared in Supplementary Table S1.

### 2.2. Anthropometric and Biochemical Measurements

The collection and measurement of anthropometric, cardiometabolic, and lifestyle-related data have been described in detail before [13,15,30]. Whole blood samples were collected for genotyping and serum biomarker measurements after an overnight fast [13,15,30].

Serum AMH levels were measured using manual enzyme-linked immunosorbent assays (ELISA) from Ansh Labs (Webster, TX). In the 2002–03 family study, AMH levels were determined for 198 participants using the picoAMH ELISA assay; AMH levels for 14 women were determined with the Ultrasensitive (us)AMH/MIS ELISA assay. In the 2010 Soifua Manuia study, the picoAMH assay was used for women age ≥ 40 years old (*n* = 516), and the usAMH/MIS assay was used for women < 40 years old (*n* = 457) [12]. The inter- and intraassay coefficients of variation were < 15%. The detection limit was 6 pg/mL and 0.08 ng/mL for the picoAMH assay and the usAMH/MIS assay, respectively. To harmonize measurements from the two different assays, the values from the picoAMH assay were rescaled to align them with values from the usAMH/MIS assay using this equation (Ansh Lab insert AL124-i released on 27 September 2019; regression *R*^2^ = 0.99; and *p* < 0.0001):

usAMH/MIS assay (ng/mL)=(picoAMH assay (pg/mL)+50.66) / 0.92 / 1000 

### 2.3. Genotyping and Imputation

Genotyping in the 2002–03 family study was performed using the Global Screening Array-24 v.3.0 BeadChip (Illumina, CA, USA) with 644,880 SNVs including custom content pertinent to Samoans. For the 2010 Soifua Manuia study, 659,492 SNVs were genotyped genome-wide using Affymetrix 6.0 array. Quality control procedures were implemented for genotypes from both arrays following the guidelines outlined by Laurie et al. (2010). Detailed description of genotyping and quality control have been previously published [15,30]. 

Using a reference panel derived from 1285 Samoan individuals with whole-genome sequencing [31], we performed imputation using minimac4 in both samples and removed variants with *R*^2^ < 0.3, yielding an additional 16,744,117 (2002–03 family study) and 15,633,124 (2010 Soifua Manuia study) SNVs [32].

### 2.4. Ethical Approval

Research protocols, informed consents, and secondary analyses of both studies were approved by the Health Research Committee of the Samoan Ministry of Health and the Institutional Review Board of Brown University for the 2010 Soifua Manuia study and additionally the American Samoa Department of Health IRB for the 2002–03 family study [13,15,30]. All participants were informed about their rights verbally in Samoan by trained research staff before obtaining their written consent [13,15,30].

### 2.5. Genome-Wide Association Study

To account for AMH levels below the detection limit (2002–03 family study: *n* = 1; 2010 Soifua Manuia study: *n* = 169), we tested for association between genotype dosages and AMH levels using a Cox mixed-effects model as implemented in the R package {coxmeg} [33,34]. Since Cox regression is designed for right-truncated data, we used the reciprocal of the measured AMH levels [35]. We adjusted for fixed effects of centered age and centered age^2^ (as well as polity for the 2002–03 family study) and for random effects of genetic relatedness using empirical kinship coefficients as estimated by PC-Relate [36]. Due to the genetic homogeneity of the sample [15], we did not adjust for principal components of ancestry.

The association results from both Samoan samples were meta-analysed using a *p* value–based fixed-effect approach via METAL [37]. Before combining the two association studies, results from the 2002–03 family study and the 2010 Soifua Manuia study were filtered for minor allele frequency (MAF) ≥ 0.05 and MAF ≥ 0.01, respectively, to keep the minor allele count between the two samples close in range. SNVs with a *p* value ≤ 1 × 10^−5^ in the two pre-meta-analysis GWASs were tested for goodness of fit to Hardy–Weinberg equilibrium, and SNVs with *p* < 0.0001 were excluded from subsequent analyses. Conditional analyses to detect secondary signals were conducted by including the lead SNVs in each suggestively associated region as covariates in the original model.

Manhattan and QQ plots were created using the R package {fastman} [38]. Tests with *p* values ≤ 5 × 10^−8^ were considered genome-wide significant, and ≤1 × 10^−5^ were considered suggestive. Genomic positions are in Genome Reference Consortium Human Build 38 (hg38).

To refine the regions of associations and determine the most probable causal variant, each locus with a *p* value ≤ 1 × 10^−5^ was visualized with LocusZoom [39] and assigned a posterior probability via Bayesian fine-mapping with PAINTOR v2.1. [40] using functional annotation from the Ensembl Variant Effect Predictor (VEP, [41]) and RegulomeDB [42]. This fine-mapping was carried out in a region ±500 kb around each lead SNV, accounting for Samoan-specific linkage disequilibrium (LD) structure.

To identify gene sets and pathways that are enriched for variants relevant to AMH variation, GWAS meta-analysis summary statistics were processed by the Functional Mapping and Annotation (FUMA) v1.5.2 genetic associations pipeline [43] after converting the genomic positions from UCSC hg38 to genomic build UCSC hg19 via liftOver [44]. In addition to functional annotation, we used FUMA to carry out gene-based analysis via Multi-marker Analysis of GenoMic Annotation (MAGMA) [45] and preliminary expression quantitative trait loci (eQTL) analysis. Independent loci were sets of SNVs with a GWAS *p* value ≤ 1 × 10^−5^ and not in high LD (*r*^2^ < 0.6) with other SNVs with *p* value ≤ 1 × 10^−5^ on the same chromosome. Candidate SNVs for examination with FUMA were SNVs within ±500 kb of the lead SNV that had a GWAS *p* value < 0.05 and were in LD (*r*^2^ ≥ 0.6) with the lead SNVs. LD structure for FUMA analysis was calculated using all populations of the 1000G phase 3. Variants located outside the gene boundaries but relevant to the nearby gene are assigned to it by MAGMA. The annotation window size for proxy SNVs was set at 40 kb upstream and 10 kb downstream around each gene. The significance threshold for the gene-based test was set at *p* = 2.50 × 10^−6^ following Bonferroni correction for 20,000 tests. eQTL analysis interrogated whole blood, endocrine tissues (adrenal gland, hypothalamus, ovary, pancreas, pituitary, and thyroid) and metabolic tissues (subcutaneous adipose tissue and liver) using data from GTEx v8 [46]. *p* values in the eQTL analyses were Bonferroni-corrected based on the number of genes assessed.

### 2.6. Known AMH Loci

We examined our GWAS summary statistics for evidence of association of AMH levels with the reported lead SNVs in the eight known AMH loci (*TEX41* [2-144887307-A-G] [21,23]; *CDCA7* [2-173394597-C-T] [21]; *CTB-99A3.1* [5-146560687-G-A] [21]; *EIF4EBP1* [8-38015258-C-T] [23]; *BMP4* [14-53956049-G-T] [23]; *AMH* [19-2251818-T-C] [23]; *MCM8* [20-5967581-G-A] [20,21,23]; and *CHECK2* [22-28707610-T-C] [23]). A locus was considered replicated if the lead SNV was present in the meta-analysis, had a *p* value < 0.05, and had the same effect direction.

When the lead SNVs from these prior GWASs was absent from the meta-analysis, because the allele frequency was too low for inclusion in the meta-analysis, we also report the lead SNV within ±50 kb of the prior GWAS’s lead SNV if it had *p* < 0.05. For each of these nearby SNVs we also calculated a per-region Bonferroni-corrected significance threshold. The threshold was corrected for the number of independent SNVs in the region as calculated by simpleM [47].

### 2.7. Transcriptome-Wide Association Study

To assess the association of estimated gene expression levels based on genotypes with the phenotypes, we carried out a transcriptome-wide association study (TWAS) on meta-analysis summary statistics with the MetaXcan [48,49,50] suite of tools. We used the MASHR algorithm to predict gene expression, as it has demonstrated better performance than the Elastic Net algorithm [51]. We performed TWAS in whole blood, endocrine tissues (adrenal gland, hypothalamus, ovary, pancreas, pituitary, and thyroid) and metabolic tissues (liver and subcutaneous adipose tissue) using S-PrediXcan and combined the results for each gene from all tested single-tissue models into a single aggregate statistic using S-MultiXcan [49]. The *p* value for statistical significance (*p =* 2.61 × 10^−6^) is adjusted for the number of genes tested (*n* = 19,158).

### 2.8. Colocalization Analysis

Colocalization analyses were performed using fastENLOC [52,53,54,55]. For this, we converted the *z* scores from the meta-analysis to posterior inclusion probabilities (PIP) for causality via TORUS and colocalized 5,589,187 variants with eQTL data from GTEx within the nine issues listed above [56]. Precomputed GTEx multi-tissue annotations are available at https://github.com/xqwen/fastenloc (accessed on 21 December 2022) [52,53,54,55]. 

## 3. Results

The socio-demographic characteristics and AMH levels for the two samples are presented in Table 1, and AMH levels by age are depicted in Supplementary Figure S2.

**Figure 1:**
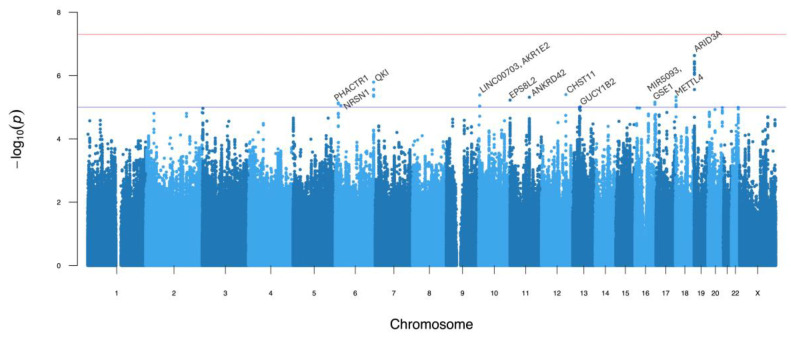
Manhattan plot for AMH levels. The solid red and blue lines denote the genome-wide significant and suggestive p value thresholds at *p* < 5 × 10^−8^ and *p* < 1 × 10^−5^, respectively. The peak SNV in each independent locus that surpassed the suggestive threshold is labeled with the nearby genes.

The GWAS of the AMH levels had no genome-wide statistically significant associations (*p* ≤ 5 × 10^−8^), but eleven loci with *p* ≤ 1 × 10^−5^ were observed (Figure 1, Table 2, and Supplementary Table S2). The quantile–quantile plot (Supplementary Figure S3) shows no evidence of genomic inflation of the test statistics (genomic inflation factor λ_GC_  =  1.09). The lead variants of each locus also had the highest posterior probability after fine-mapping for causal variants, except for the lead variant in *ARID3A* (Table 2). The quality score of all imputed lead variants were >0.88. We confirmed via conditional analysis that no secondary signals were present in suggestive loci, for each of the suggestive loci (regional plots in Supplementary Figure S4).

We replicated one of the eight known AMH loci: *EIF4EBP1* (Supplementary Table S3, Supplementary Figures S7 and S8). Of the seven known AMH loci with lead variants not replicated here, the lead variants in three (*MCM8*, *CHECK2*, and *CTB99A3.1*) were ultra-rare in Samoans (MAF < 0.0001). Within ±50 kb of two unreplicated loci (*AMH* and *TEX41*), we detected two SNVs with a significant *p*-value (2-144839456-A-T [*p* = 0.0018] and 19-2250470-G-A [*p* = 0.0006], respectively); however, they were not in LD with the known AMH loci.

**Figure 2:**
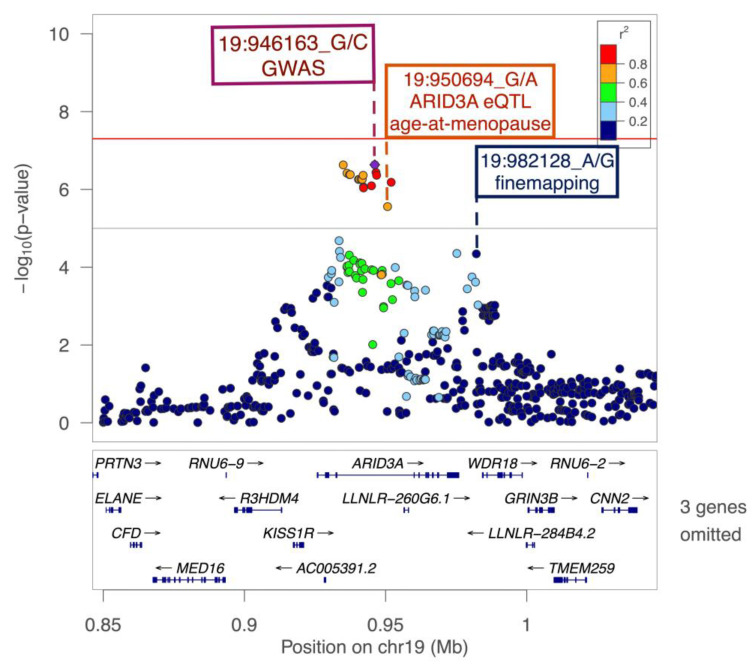
Regional plot for ARID3A locus. 19-946163-G-C, the lead SNV, is a purple diamond. The color of all other SNVs reflects LD with the lead SNV as calculated in the Samoan samples. The solid red and grey line indicate the significance and suggestive thresholds at *p* < 5 × 10^−8^ and *p* < 1 × 10^−5^, respectively.

The strongest GWAS association is in intron 3 of *ARID3A* at 19p13.3. The lead SNV, 19-946163-G-C (*p* = 2.32 × 10^−7^), and nearby SNVs are presented in Figure 2. The alternate allele of the lead SNV was associated with lower AMH levels (Supplementary Figures S5 and S6). This locus also harbors the known age-at-menopause variant 19-950694-G-A, observed in women of European ancestry, which is in high LD (*r*^2^ = 0.79) with the lead AMH variant in this locus in this study [57,58]. Fine-mapping identified 19-982128-A-G, upstream of *WDR18*, as the most probable causal variant with a posterior probability (PP) of 0.34. The lead AMH GWAS variant, 19-946163-G-C (PP = 0.11), and age-at-menopause variant 19-950694-G-A (PP = 0.02) were the top two eQTLs affecting *ARID3A* expression in the thyroid tissue (*p* = 5.75 × 10^−7^ and *p* = 3.69 × 10^−7^, respectively, both with an FDR = 2.60 × 10^−10^).

Gene-based analysis identified five significant associations with AMH levels: *ARID3A* (*p =* 5 × 10^−10^) and nearby *R3HDM4* (*p =* 1.47 × 10^−9^) as well as *P2RX6* (*p =* 1.15 × 10^−6^), *AC002472.1* (*p =* 8.30 × 10^−7^), and *PTPRB* (*p =* 2.47× 10^−6^) (Supplementary Figure S9).

**Figure 3:**
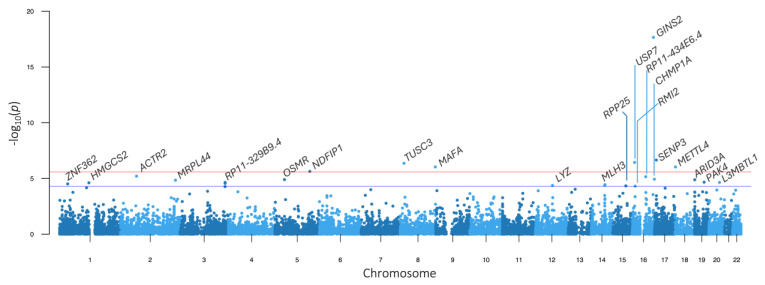
Manhattan plot of TWAS results for AMH. The solid red and blue lines denote the transcriptome-wide significant and suggestive *p* value thresholds at *p* < 2.6 × 10^−6^ and *p* < 1 × 10^−5^, respectively. The genes that surpassed the suggestive threshold are annotated.

There were seven transcriptome-wide significant genes observed in the TWAS of whole blood, endocrine tissues (adrenal gland, hypothalamus, ovary, pancreas, pituitary, and thyroid), and metabolic tissues (liver and subcutaneous adipose tissue) in association with AMH levels. The strongest association of the seven was *GINS2*. *METTL4* was not only transcriptome-wide significant but also suggestively associated with AMH levels. The TWAS results are presented in Figure 3 and Supplementary Table S4.

We conducted a colocalization analysis using summary statistics from the GWAS and TWAS. In SNV-level and gene-level colocalization analysis, *ARID3A* had a locus-level colocalization probability (LCP) of 0.34 in the liver. All other colocalization probabilities for other genes and tissues were below the threshold of 0.30.

## 4. Discussion

Here we report the first study examining association between genetic variants and AMH levels in women from a Polynesian population. We identified eleven novel suggestive loci via GWAS (*ARID3A*, *QKI*, *CHST11*, *AKR1E2*, *METTL4*, *ANKRD42*, *EPS8L2*, *GSE1*, *EDN1*, *NRSN1*, and *GUCY1B2*) and seven significant genes via TWAS (*GINS2*, *SENP3*, *USP7*, *TUSC3*, *MAFA*, *METTL4*, and *NDFIP1*). Additionally, we replicated the known AMH locus *EIF4EBP1* previously detected in women of European ancestry [23]. Among these findings, three genomic risk loci—*ARID3A*, *GSE1* and nearby *GINS2*, and *METTL4*—had associations in both GWAS and TWAS and are biologically plausible. 

Our key GWAS finding was the association of AMH with variants in the gene encoding AT-rich interaction domain 3A (*ARID3A*), a member of a family of proteins regulating chromatin binding. It was also a significant locus in gene-based analysis and was suggestively significant in the TWAS. The lead variant in *ARID3A* is located 4.5 kb upstream from a known age-at-menopause variant [57,58], and the two are in high LD. The biological link between AMH levels (a marker of ovarian follicle reserve) and age-at menopause is well recognized, as AMH testing is used to predict time-to-menopause in late-reproductive-age women in clinical practice [59,60]. Notably, both the lead *ARID3A* GWAS variant and the known age-at-menopause variant are positioned within GeneHancer [61] regulatory element GH19J000942, a target of which is the gene encoding KISS1 (kisspeptin) receptor (*KISS1R*). While there is not strong statistical evidence of gene-based or TWAS associations for *KISS1R* itself in this study, that does not rule it out as having a causal role in this locus. Kisspeptin/KISS1R signaling is pivotal in folliculogenesis [62]. Increased ovarian kisspeptin levels hinder the transition of primary follicle into antral stage by inhibiting the *FSHR* expression and increasing the AMH levels [62]. Observations in mice with conditional ablation of *Kiss1r* in oocytes suggest that the resulting deregulation may lead to premature ovarian failure [63]. Although the lead variant is in an intron of *ARID3A*, should this variant be causal or in LD with the causal variant, it may be acting through an effect on *KISS1R*. 

In the TWAS, *GINS2* was the most significant gene and is within the *GSE1* locus on 16q24.1, which was suggestively significant in the GWAS. *GINS2* is highly conserved among eukaryotes and encodes one of the essential subunits that form the tetrameric Go-Ichi-Nii-San (GINS) complex, which has a key role in DNA replication [64,65]. *GINS2* is downregulated in ovaries of old-aged rhesus monkeys compared to young- and middle-aged ones and in atretic bovine ovarian follicles compared to the healthy ones [66,67]. *GSE1*, an epigenetic regulator and a known oncogene, may also have a biological connection to AMH levels, as it has highest expression in the pituitary and the ovary [46,68]. The expression of *Gse1* in the primordial follicles is downregulated in estrogen receptor β (ERβ) knockout mice [69]. Loss of ERβ activates follicle growth and leads to early depletion of the ovary reserve, suggesting a regulatory role for *Gse1* in folliculogenesis [69]. AMH stimulates gonadotropin-releasing hormone (GnRH) expression in its role in the hypothalamic–pituitary–gonadal hormonal axis, and therefore, it is notable that *Gse1* is highly enriched in GnRH neurons upon gonadectomy [70,71].

The *METTL4* locus on 18p11.32 includes *METTL4* and *NDC80* and was identified by both GWAS and TWAS analyses (Supplementary Figure S4J). *METTL4* encodes methyltransferase 4, which is responsible for the adenine methylation involved in regulating RNA-splicing [72]. Epigenetic regulation via N^6^-methyladenosine modifications plays an active role in response to environmental stressors (hypoxia, starvation, toxicants, etc.) and has been reported to be a relevant mechanism in the development of PCOS and premature ovarian insufficiency [73,74,75,76,77]. While *METTL3*, a paralog of *METTL4*, has been implicated in follicle development and fertility by regulating the stability of oocyte meiotic maturation-related transcripts in mice, not much is known about the role of *METTL4* in female reproduction [72,78,79]. Nearby *NDC80* encodes a component of the nuclear division cycle 80 kinetochore complex [80,81]. In mouse oocytes, this complex partakes in initiating oocyte maturation by enabling the spindle assembly required for G2/M transition by stabilizing cyclin B2 levels [81,82,83,84,85]. Consequently, the dormant primordial follicles arrested since birth at G2/M resume meiosis [81,82,83,84]. Association of variants near *METTL4* may indicate that genes that control the G2/M transition have a biological connection to modulation AMH levels.

We replicated one of the eight known AMH loci—intergenic variant 8-38015258-C-T (rs10093345) near *EIF4EBP1*. This SNV was also significantly associated with age-at-menopause in the UK Biobank [86]. Notably, lead variants in four known AMH loci—*AMH*, *CHECK2*, *CTB-99A3.1*, and *MCM8*—are low-frequency or rare variants in Europeans; these same variants in Samoans are either rare with low Samoan-specific imputation quality (*R*^2^ < 0.10) or are ultra-rare (MAF < 0.0007). Such population-specific susceptibility loci highlight differences in AMH genetic architecture between Samoan individuals and individuals of European ancestry and highlight the importance of diversifying study populations. Recently, Moolhuijsen et al. investigated whether *AMH* promoter variation affects serum AMH levels in PCOS patients of Northern European ancestry and observed an association between rs10406324 (19-2249113-G-A) and lower AMH levels that was independent of follicle count and other PCOS markers [22]. Similar findings were also observed in normo-ovulatory women [21]. This suggests that genetic factors can contribute to variation in AMH levels independent from follicle count and/or PCOS status. In this study, rs10406324 could not be analysed due to low MAF (<0.005) and poor imputation quality (*R*^2^ < 0.10).

### Strengths and Limitations

While our sample was small compared to other published GWASs, we were uniquely positioned to identify variants that may be rare in other populations but common in Samoans due to population founder effects. Additionally, the genetic homogeneity of the Soifua Manuia [15] sample could result in better power to detect variants associated with AMH levels. 

Measuring AMH levels in older women is a challenge, as AMH levels fall below detectable ranges during perimenopause. We addressed this challenge by employing two AMH assays from Ansh Lab: picoAMH and ultra-sensitive AMH/MIS ELISA kits for older women and younger women, respectively. These assays utilize the same antibodies and calibrators, with picoAMH extending coverage for the lower ranges of the standard curve [87]. To reduce the heterogeneity, we used the conversion factor from Ansh Lab to harmonize the AMH levels between the two age groups. Additionally, we robustly accounted for the AMH values below detectable limits in our GWASs by leveraging the Cox’s proportional hazards regression model via coxmeg. 

For GWAS findings, although we cannot entirely rule out the possibility of false positive (FP) results and acknowledge that a plausible story for those can be made easily [88], we aimed to mitigate this by prioritizing the findings with evidence from both GWAS and TWAS analyses. Furthermore, while we identified several significant TWAS associations, absence of Samoan-specific eQTLs and limited representation of diverse populations in GTEx could have led to reduced power and detection of fewer eQTL associations.

## 5. Conclusions

Overall, eleven loci were suggestively associated with AMH levels in a meta-analysis of Samoan women, several of which have plausible links to ovarian function/folliculogenesis (*KISS1R*, *GINS2*, and *NDC80*). This study provides valuable insights into the genetic variation of AMH in Samoan women and replicates the previously detected association of *EIF4BP1* with AMH levels. The putative novel findings in this study will need to be validated in additional larger studies. The identification of variations affecting AMH levels, such as seen in our findings, may also improve understanding of the biological underpinnings of AMH-related reproductive traits such as ovarian function, age at menopause, premature ovarian failure, and PCOS. Eventually, these findings may contribute to the development of screening tools measuring the genetic susceptibility for AMH-related traits.

## Figures and Tables

**Table 1:** Sample demographics and characteristics for quantitative traits.

	2002–03 Family Study						2010 Soifua Manuia Study					
Variable	*n*	Mean	sd	Min	Median	Max	*N*	Mean	sd	Min	Median	Max
Age	212	28.3	6.8	18.0	28.1	39.8	973	39.3	7.6	25.0	40.7	50.9
BMI (kg/m^2^)	212	34.0	8.5	20.4	32.9	69.0	971	34.8	6.8	18.0	34.4	59.9
AMH (ng/mL) ^†^	212	3.90	6.01	0.06	2.81	77.5	973	1.64	2.65	0.06	0.59	25.8
AMH (ng/mL) ^††^	211	3.91	6.02	0.08	2.82	77.5	804	1.97	2.81	0.06	0.97	25.8
Polity American Samoa	60%						0%					
Samoa	40%						100%					

^†^ values below the assay limit of detection are winsorized to the detection limit values. ^††^ values below the assay limit of detection excluded.

**Table 2:** Meta-analysis results for AMH levels presenting independent SNVs with *p* value ≤ 1 × 10^−5^.

Locus Information						2002–03 Family Study		2010 Soifua Manuia Study		Meta- Analysis	
Lead Variant	Nearest Gene	Type	RDB	Samoan EAF	EUR EAF	β (SE)	*p*	β (SE)	*p*	*p*	PP
19-946163-G-C	*ARID3A*	intronic	5	0.453	0.123	−0.13 (0.13)	3.10 × 10^−1^	−0.39 (0.08)	1.67 × 10^−7^	2.32 × 10^−7^	0.11
6-163620593-G-A	*QKI*	intergenic	5	0.132	*	−0.14 (0.18)	4.27 × 10^−1^	−0.57 (0.12)	8.52 × 10^−7^	1.61 × 10^−6^	0.67
12-104584595-T-A	*CHST11*	intronic	6	0.845	0.524	−0.32 (0.18)	6.78 × 10^−2^	−0.44 (0.10)	2.26 × 10−5	3.98 × 10^−6^	0.63
10-4474887-C-A	*AKR1E2*	intergenic	5	0.069	*	0.48 (0.24)	4.49 × 10^−2^	0.63 (0.15)	3.36 × 10^−5^	4.09 × 10^−6^	0.40
18-2490805-C-T	*METTL4*	intergenic	7	0.787	0.346	−0.20 (0.15)	1.83 × 10^−1^	−0.42 (0.09)	9.46 × 10^−6^	4.73 × 10^−6^	0.34
11-83219203-T-C	*ANKRD42*	intronic	4	0.285	0.347	−0.38 (0.14)	6.55 × 10^−3^	−0.31 (0.08)	1.59 × 10^−4^	4.83 × 10^−6^	0.06
11-722202-G-C	*EPS8L2*	intronic	3a	0.412	0.194	0.33 (0.14)	1.57 × 10^−2^	0.29 (0.08)	1.09 × 10^−4^	5.96 × 10^−6^	0.33
16-85420473-G-A	*GSE1*	intergenic	5	0.877	0.318	−0.27 (0.20)	1.87 × 10^−1^	−0.48 (0.11)	1.39 × 10^−5^	6.91 × 10^−6^	0.59
6-12525440-G-A	*PHACTR1*	intergenic	7	0.097	0.119	0.09 (0.21)	6.78 × 10^−1^	−0.67 (0.13)	2.71 × 10^−7^	7.32 × 10^−6^	0.17
6-23537402-C-G	*NRSN1*	intergenic	4	0.052	0.000	0.31 (0.26)	2.43 × 10^−1^	0.82 (0.19)	1.31 × 10^−5^	8.87 × 10^−6^	0.77
13-51023905-C-T	*GUCY1B2*	intronic	7	0.117	0.279	0.79 (0.20)	5.73 × 10^−5^	0.35 (0.12)	2.70 × 10^−3^	9.84 × 10^−6^	0.36

Each variant is presented with its gnomAD ID. We reported the effect allele (EA), its frequency (EAF), and the effect estimates (β) with a standard error (SE). The EUR EAF reports the effect allele frequency in Europeans observed in the 1000 Genome project as reported by gnomAD v4 [37]. The asterisk (*) indicates that the variant is unobserved in 1000G. *R*^2^ is the imputation quality in non-genotyped markers. RDB is the RegulomeDB functional class, with class scores ≤ 2 having greater probability of a transcriptional regulatory role. The posterior probability (PP) was calculated using PAINTOR.

## Data Availability

Genotype and phenotype data for 2010 Soifua Manuia study are avail-able in dbGAP under accession numbers phs000914 and phs000972. Genotype and phenotype data for the 2002–03 family study are not available due to consent restrictions.

## Supplementary Materials

The following supporting information can be downloaded at https://www.mdpi.com/article/10.3390/genes16070793/s1. Supplementary Materials include Supplementary Figures (Figures S1–S9) and Supplementary Tables (Tables S1–S4).

## Abbreviations

The following abbreviations are used in this manuscript:

AMH	Anti-Müllerian hormone
eQTL	Expression quantitative trait locus
GWAS	Genome-wide association study
LD	Linkage disequilibrium
MAF	Minor allele frequency
TWAS	Transcriptome-wide association study
