# Supplementary material for: A Genome-Wide Association Study of Anti-Müllerian Hormone (AMH) Levels in Samoan Women
Source: Genes (Basel). 2025 Jun 30;16(7):793. doi: 10.3390/genes16070793 (PMC12294779; doi:10.3390/genes16070793)
Supplement: Supplementary file 1 [file genes-16-00793-s001.zip › genes-3733199-supplementary.pdf]

A genome-wide association study of anti-Müllerian hormone levels in Samoan women

Supplementary Material

Erdogan-Yildirim Z, Carlson JC, Krishnan M, Zhang JZ, Lambert-Messerlian G, Naseri T, Viali S, Hawley NL, McGarvey ST, Weeks DE, Minster RL

Content

Abstract in Gagana Sāmoa 2

Supplementary Figure 1 Flow chart diagrams showing the sample selection for Study Sample 1 (left) and for Study Sample 2 (right) 4
Supplementary Figure 2. AMH levels by age in both samples. 5
Supplementary Figure 3. Quantile–quantile plot for AMH genome-wide association meta-analysis 5
Supplementary Figure 4. Regional plots for genome-wide association on AMH 6
Supplementary Figure 5. AMH levels for the lead variant 19-946163-G-C at *ARID3A* in 2002–03 Family Study (top) and 2010 Soifua Manuia Study (bottom) 9
Supplementary Figure 6. AMH levels by genotype strata for 2002–03 Family Study (top) and 2010 Soifua Manuia Study (bottom) using survival curve 10
Supplementary Figure 7. AMH levels for the lead variant 8-38015258-C-T at *EIF4EBP1* in 2002–03 Family Study (top) and 2010 Soifua Manuia Study (bottom) 11
Supplementary Figure 8. AMH levels by genotype strata for 2002–03 Family Study (top) and 2010 Soifua Manuia Study (bottom) using survival curve 12
Supplementary Figure 9. Manhattan plot of the gene-based test of meta-analysis summary statistics via MAGMA implemented in FUMA 13

Supplementary Table 1 Statistical comparison of baseline characteristics between individuals with measured and unmeasured serum AMH levels 4
Supplementary Table 2 GWAS results with *p* values < 1 × 10⁻⁵ 14
Supplementary Table 3 Results from transcriptome-wide analysis 16
Supplementary Table 4 Look-up of known AMH loci in Samoan GWAS 17

ABSTRACT IN GAGANA SĀMOA

O le saʻiliʻiliga ile ʻeseʻesega o tupuʻaga ma ona aʻafiaga i le tulaga o homone poʻo le aofai o fuāmoa/fua fanau ile itupa o tamaʻitaʻi o loʻo totoe i totonu o le ovari i tamaʻitaʻi o Samoa

Faʻamatalaga

Fesili mo suʻesuʻega:
E mafai ona suʻesuʻeina le eseʻesega o tupuʻaga o kenera (GWAS), ma le maualuga o gaioiga o kenera (TWAS) ma fesoasoani ia iloa le eseesega o kenera e fesoʻotaʻi ma le taʻamilosaga, ma le aofaʻiga o fua fanau o le homone (AMH) i fafine Samoa.

Aotelega o tali:
Na matou faʻailoaina itulagi e sefulutasi e ala i le GWAS (faʻalapotopotoga e pito sili ona malosi i kenera *ARID3A* 19:946163_G/C [$p = 2.32 \times 10^{-7}$]) ma kenera e fitu e ala i le TWAS (*GINS2*, *SENP3*, *USP7*, *TUSC3*, *MAFA*, *METTL4*, *NDFIP1* [o mea uma le $p < 2.50 \times 10^{-6}$]) faʻasalalau faʻatasi AMH maualuga i tamaʻitai Samoa.

O a mataupu ua faʻailoa:
E tolu GWASs i tausaga taʻitasi ua maua i itulagi e valu i le DNA e fesoʻotaʻi ma tulaga AMH i fafine o tupuʻaga Europa (*AMH*, *MCM8*, *TEX41*, *CHECK2*, *CDCA7*, *EIF4EBP1*, *BMP4* ma le *CTB-99A3.1*). O le *MCM8* na vaʻaia i suʻesuʻega uma e tolu.

Mamanu o le suʻesuʻega, o le telē ma le umi:
E iai la matou faʻataʻitaʻiga e tusa ma le 1,185 tina mai Samoa ma Amerika Samoa i le GWAS mai le tuʻuʻeseʻeseina o faʻataʻitaʻiga laiti e lua: Suʻesuʻega Soʻotaga faʻa-le-aiga (n = 212; 18 i le 48 tausaga) faʻatasi ai ma le mamanu o suʻesuʻega faʻa-le-aiga, o i latou uma sa resitalaina i le tausaga 2002/2003, ma le Suʻesuʻega o le Soifua Manuia (n = 973; 25 i le 51 tausaga),o se faitau aofai ma se suʻesuʻega na faia i le tausaga 2010.

Tagata auai/meafaitino/metotia/faʻatulagana:
O le maualuga o le AMH o le toto na fuaina i le faʻaaogaina o le enzyme-linked immunosorbent assays (ELISA). Na matou faʻatautaia le GWAS i luga o faʻataʻitaʻiga o Samoa i vaega laiti e lua o loʻo faʻaaogaina ai le Cox e faʻamautu ai le maualuga o le AMH o tapulaʻa e mafai ona iloa ma fetuʻunaʻiga o tausaga, ma tausaga faʻatapulaʻaina, malo ma soʻotaga. O le iuga na tuʻufaʻatasia i le faʻaaogaina o se faʻataʻitaʻiga o aʻafiaga-tumau. Sa matou suʻesuʻeina le ʻeseʻesega o kenera ma $p < 1 \times 10^{-5}$ ma fuafua poʻo le ā le ʻeseʻesega o kenera i itulagi taʻitasi e mafua ai suiga i le maualuga o le AMH. Sa matou faʻaaogaina foi polokalame o le FUMA mo le faʻatinoina o le TWAS. Sa matou suʻesuʻeina foi pe afai e iai ni itulagi na ripotia faʻalilolilo i isi suʻesuʻega, na oʻo lava iai la matou suʻesuʻega.

Taunuʻuga autu, ma avanoa o matafaioi:
Na matou faʻailoaina itulagi fou e sefulutasi ($p < 1 \times 10^{-5}$) e fesoʻotaʻi ma le maualuga o le AMH, sa vaʻai ai foi *EIF4EBP1*, lipotia faʻalilolilo le kenera o le AMH i nisi o itulaga e ala i le matou GWAS. O se tasi o faʻamatalaga mataʻina i le itulagi o *ARID3A* na vaʻaia e le GWAS ma le TWAS iloiloga. O nei itulagi, e aofia ai foi ma se itulagi e iloa e aʻafia ai tausaga-i-menopause (gata ai le palapala masina), lea e fesoʻotai malosi ($r^2 = 0.79$) faʻatasi ai ma le ʻeseʻesega ole tele o kenera (19:946163:G:C) i le *ARID3A*. E le gata i lea o le

kenera o lo‘o lata ane o lo‘o fa‘apipi‘i le aoga ole ta‘amilosaga ole fanauga (*KISS1R*) fa‘ailoa mai se faiga fa‘aola, aua o le kisspeptin e pulea le atina‘eina ai sela fa‘aautagata ma e feso‘ota‘i atu i le maualuga o le AMH.

Tapula‘a, mafua‘aga o lapata‘iga:
O tapula‘a autu o la tatou su‘esu‘ega e aofia ai fa‘ata‘ita‘iga laiti mo le GWAS ma le fa‘aaogaina o se fa‘ata‘ita‘iga TWAS ua a‘oa‘oina le tele o fa‘ata‘ita‘iga i Europa e ono oo atu ai i le fa‘aitiitia le malosi fa‘afuainumera e iloa ai feso‘otaiga i le va o fa‘alavelave fa‘a-le-tagata ma le gaioiga o kenera. E mana‘omia le toe faia o la matou su‘esu‘ega i aofa‘iga tetele o tagata o le Pasefika.

‘Aafiaga lautele o fa‘amaumauga na maua:
I le va‘aia ai o itulagi e valu na maua muamua AMH loci, sa matou fa‘ailoaina ni fa‘atasiga fou. Ua iloa o lo‘o aofia ai le faitau aofa‘i o vaega fa‘avae laiti e fesoasoani e su‘e ni vaega fou e feso‘ota‘i ma le soifua maloloina. O vaega na matou maua i‘inei e mafai ona fa‘aleleia atili ai lo matou malamalama i le AMH e feso‘otai ma tulaga tau fa‘a‘autama (ovarian reserve), tausaga i tamaitai ua muta le palaplala masina, fanau le au, ma le faletonu se vaega ole fa‘a‘au tagata poo ma‘i ma fesoasoani i le fausia o ni su‘esu‘ega mo tama‘ita‘i o lo‘o lamatia i nei tulaga o lo‘o maualuga ai le AMH.

Fa‘atupeina o le su‘esu‘ega:
O lenei galuega o lo‘o lagolagoina e ala i aitalafu i le NIH R01-HL093093 (Tagata su‘esu‘e muamua: S.T.M.), R01-HL133040 (tagata su‘esu‘e muamua: R.L.M.), ma le T90-DE030853 (tagata su‘esu‘e muamua: Sfeir C). O le ripoti o le matafaioi a tusitala, ma e le‘o fai ma sui o manatu aloa‘ia o a‘oga a le atunu‘u o le soifua maloloina.

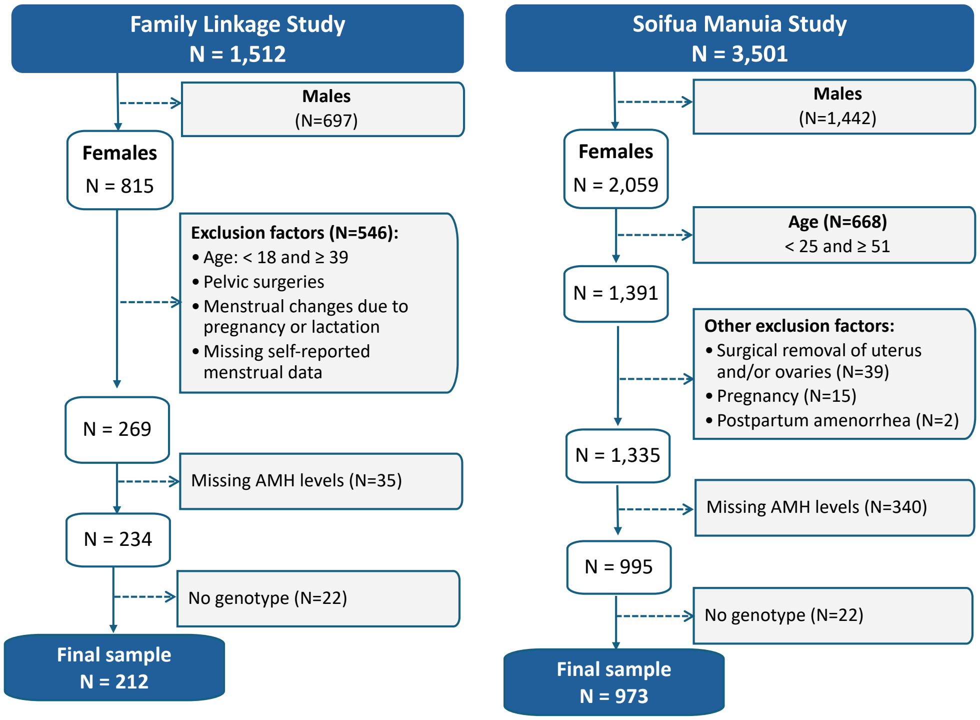

Supplementary Figure S1. Flow chart diagrams showing the sample selection for Study Sample 1 (left) and for Study Sample 2 (right).

Supplementary Table S1. Statistical comparison of baseline characteristics between individuals with measured and unmeasured serum AMH levels.

	Family Linkage Study					Soifua Manuia Study				
	AMH					AMH				
Characteristics	Unmeasured N = 35		Measured N = 234		p-value[1]	Unmeasured N = 340		Measured N = 995		p-value[1]
	N	mean (sd)	N	mean (sd)		N	mean (sd)	N	mean (sd)	
Age	35	30.26 (6.59)	234	28.41 (6.76)	0.11	340	36.23 (6.38)	995	39.25 (7.64)	<0.001
BMI	35	34.62 (6.25)	234	34.17 (8.55)	0.5	338	34.10 (5.97)	993	34.70 (6.83)	0.4
Glucose	31	85.74 (13.24)	234	90.79 (34.17)	> 0.9	191	91.32 (31.87)	942	98.80 (46.04)	0.081
Insulin	31	8.78 (9.19)	234	15.48 (19.64)	0.011	191	17.38 (14.18)	941	17.06 (17.10)	0.5
HDL	31	41.72 (9.28)	234	44.90 (9.63)	0.11	191	47.20 (11.67)	942	47.15 (11.13)	0.7
LDL	31	106.98 (27.17)	233	111.28 (29.16)	0.4	191	118.89 (28.30)	941	124.16 (30.46)	0.013
NetTG	31	124.74 (82.34)	234	104.20 (60.68)	0.4	191	97.14 (52.20)	942	106.70 (91.59)	0.049
Cholesterol	31	173.65 (30.21)	234	176.92 (32.07)	0.6	191	185.52 (32.19)	942	192.33 (33.38)	0.002

[1] Wilcoxon rank sum test

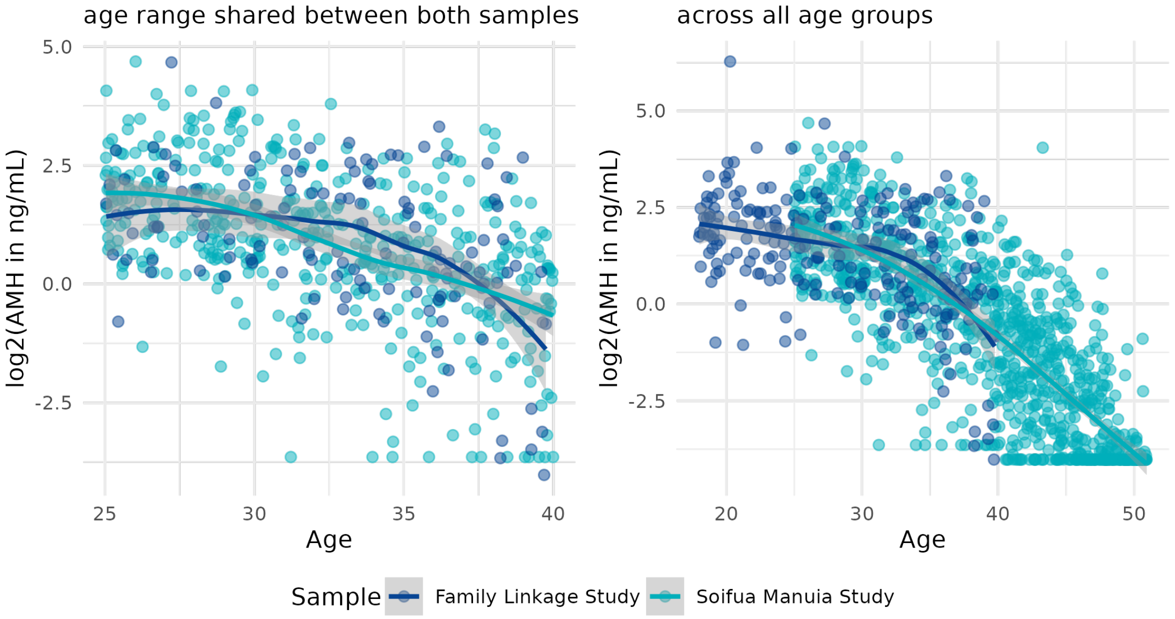

Supplementary Figure S2. AMH levels by age in both samples.

The log_2-transformed AMH levels by age are compared in overlapping (left) vs. across all (right) age ranges with smoothed loess curve.

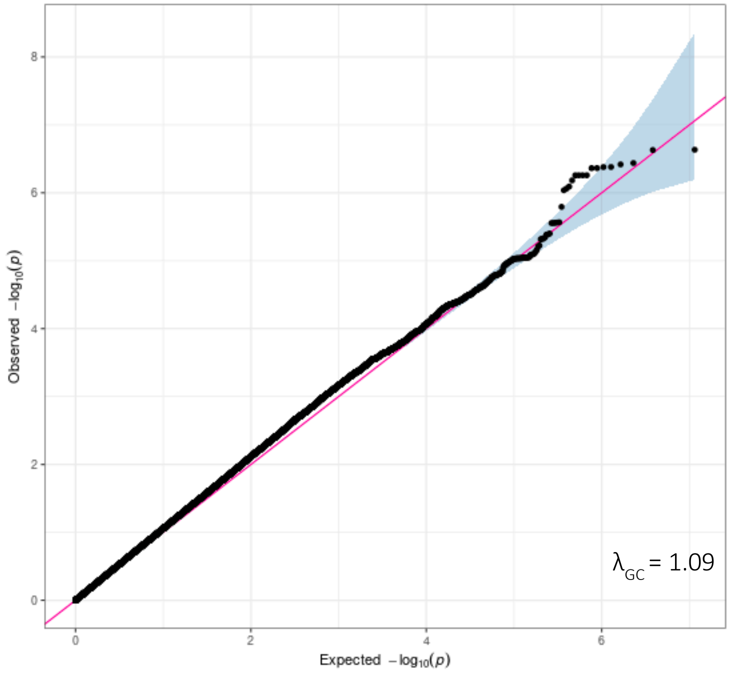

Supplementary Figure 3. Quantile–quantile plot for AMH genome-wide association meta-analysis

Regional plots for ovarian function related biomarker – AMH

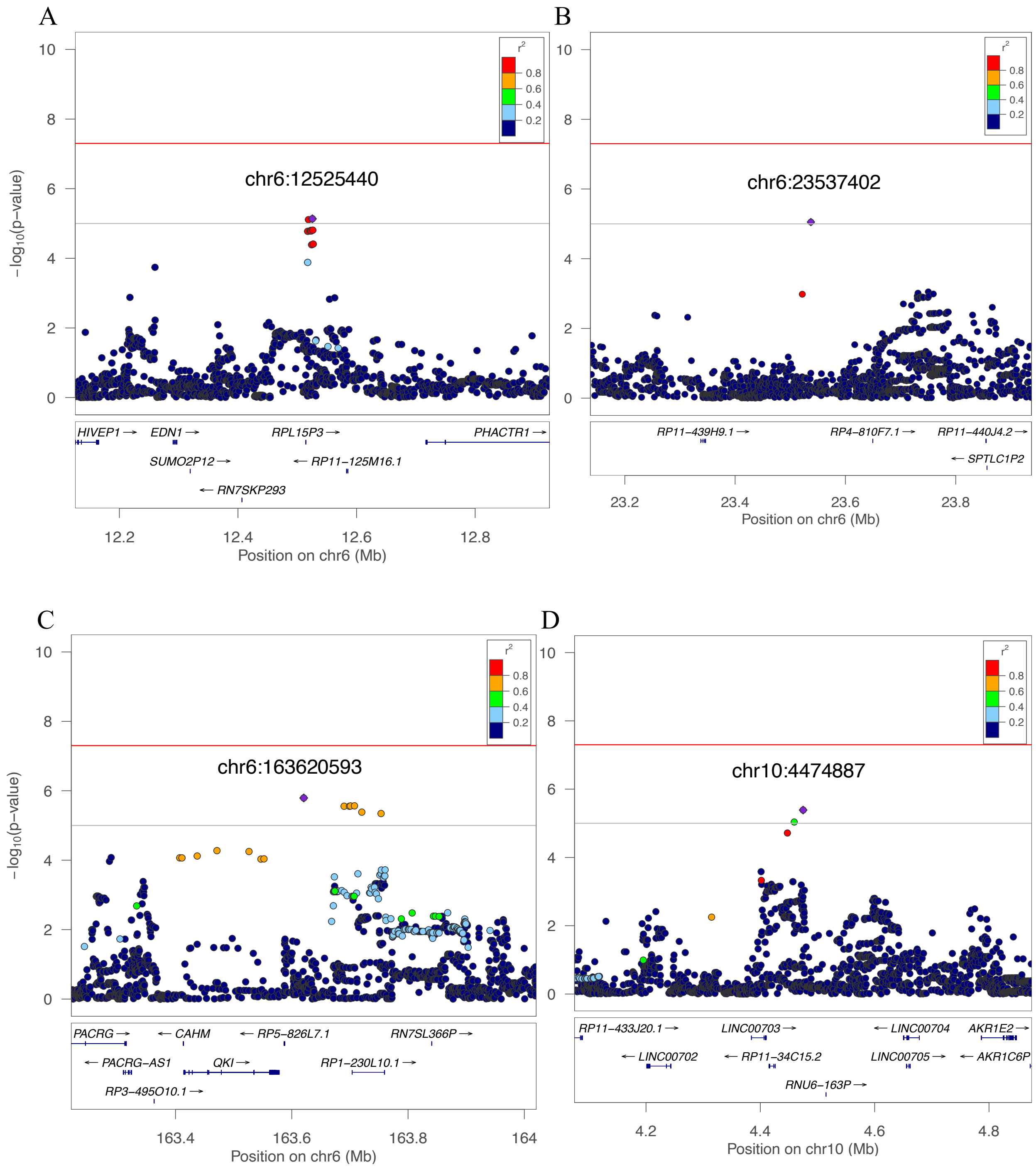

Supplementary Figure 4. Regional plots for genome-wide association on AMH

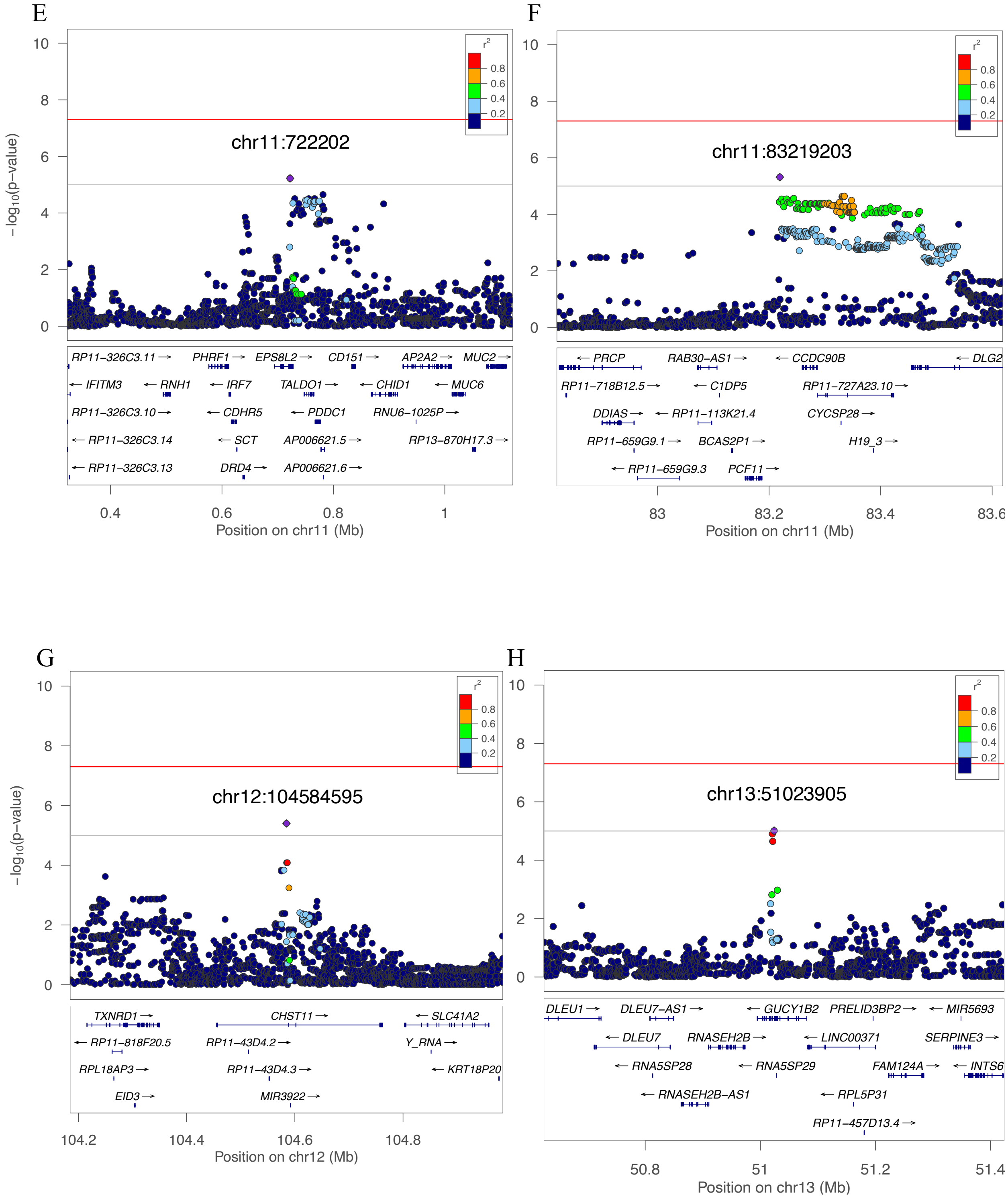

Supplementary Figure 4 (cont'd). Regional plots for genome-wide association on AMH

I

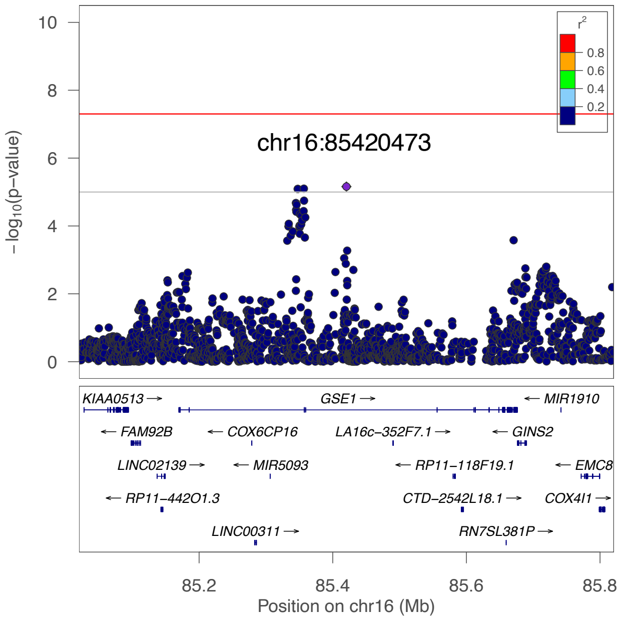

J

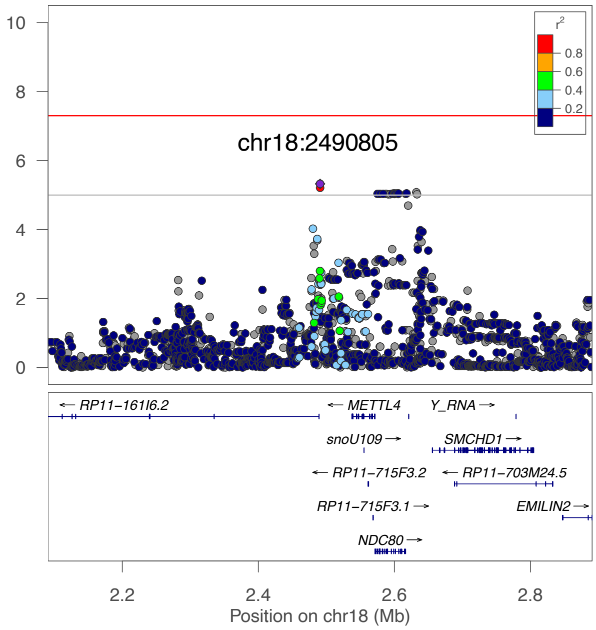

Supplementary Figure 4 (cont'd). Regional plots for genome-wide association on AMH

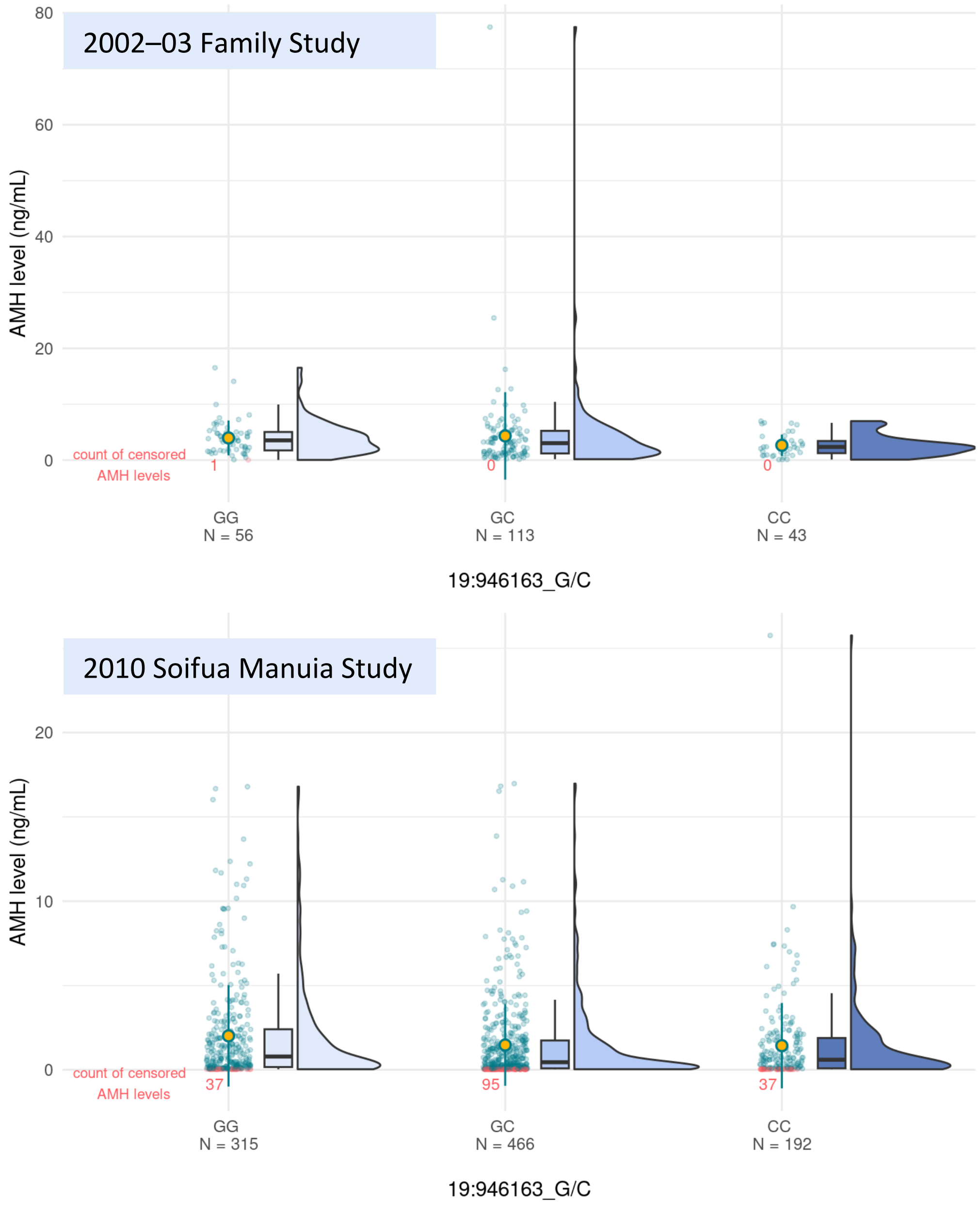

Supplementary Figure 5. AMH levels for the lead variant 19-946163-G-C at *ARID3A* in 2002–03 Family Study (top) and 2010 Soifua Manuia Study (bottom)

The mean (orange dot) and median (center line of box plot) AMH levels in ng/mL are shown by genotype. The AMH levels below detectable limits (red dots) are show with values set to the limit of detection divided by 2.

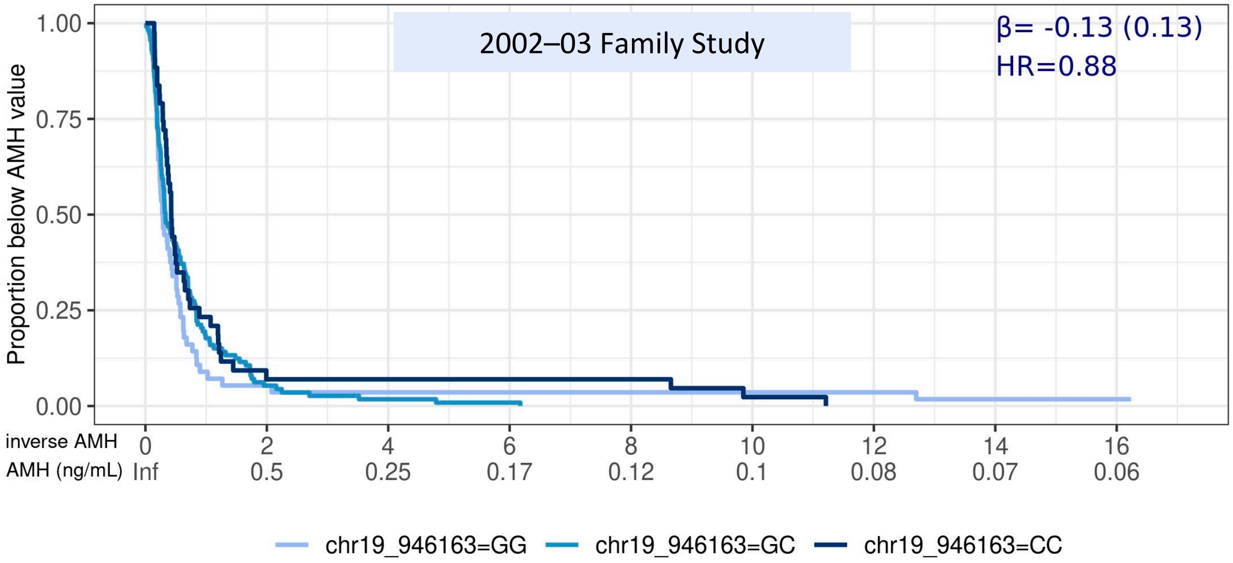

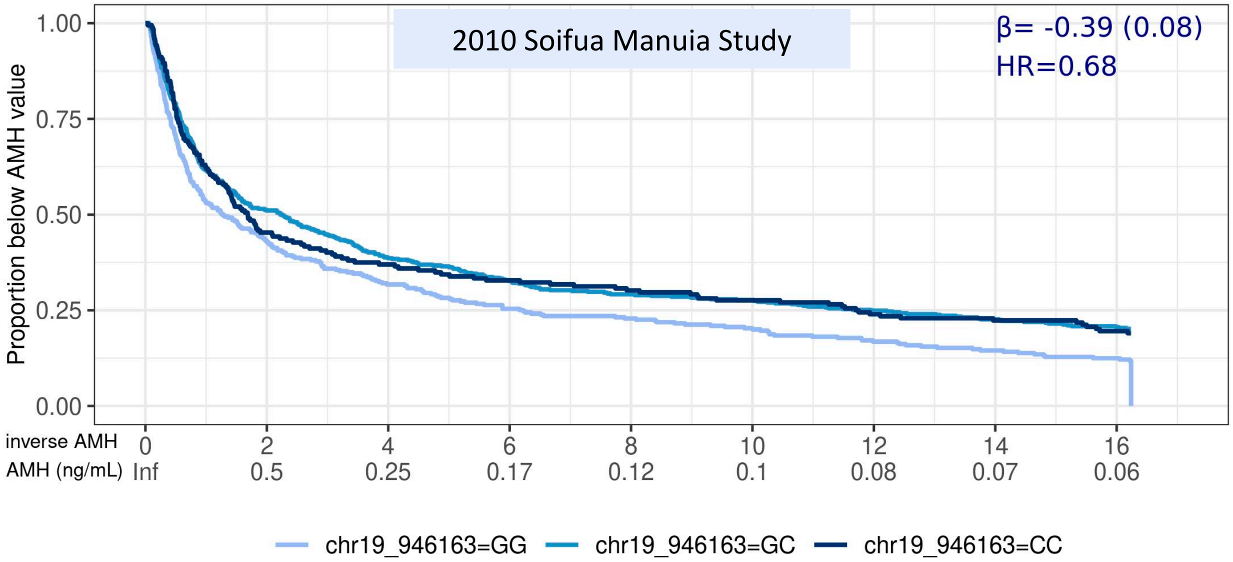

Supplementary Figure 6. AMH levels by genotype strata for 2002–03 Family Study (top) and 2010 Soifua Manuia Study (bottom) using survival curve

The inverse (or reciprocal) of the AMH level is shown in the *x* axis with the corresponding AMH levels in ng/mL beneath.

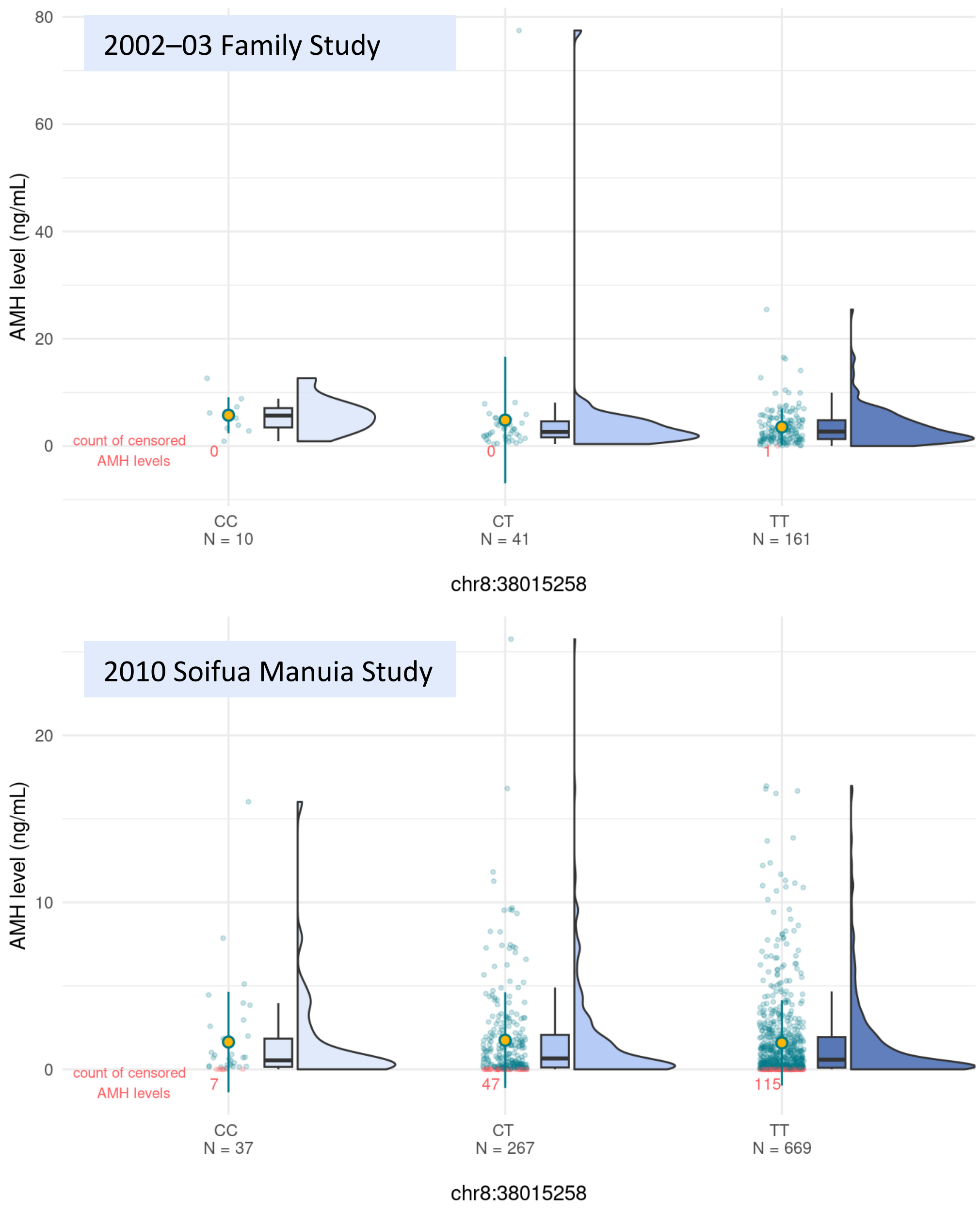

Supplementary Figure 7. AMH levels for the lead variant 8-38015258-C-T at *EIF4EBP1* in 2002–03 Family Study (top) and 2010 Soifua Manuia Study (bottom)

The mean (orange dot) and median (center line of box plot) AMH levels in ng/mL are shown by genotype. The AMH levels below detectable limits (red dots) are show with values set to the limit of detection divided by 2.

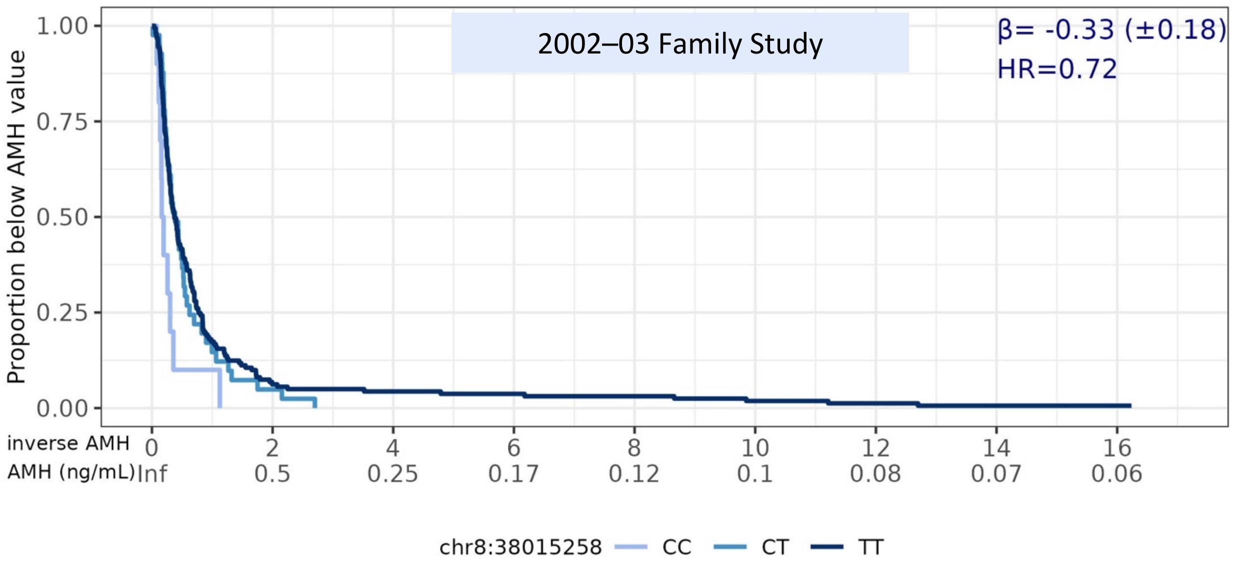

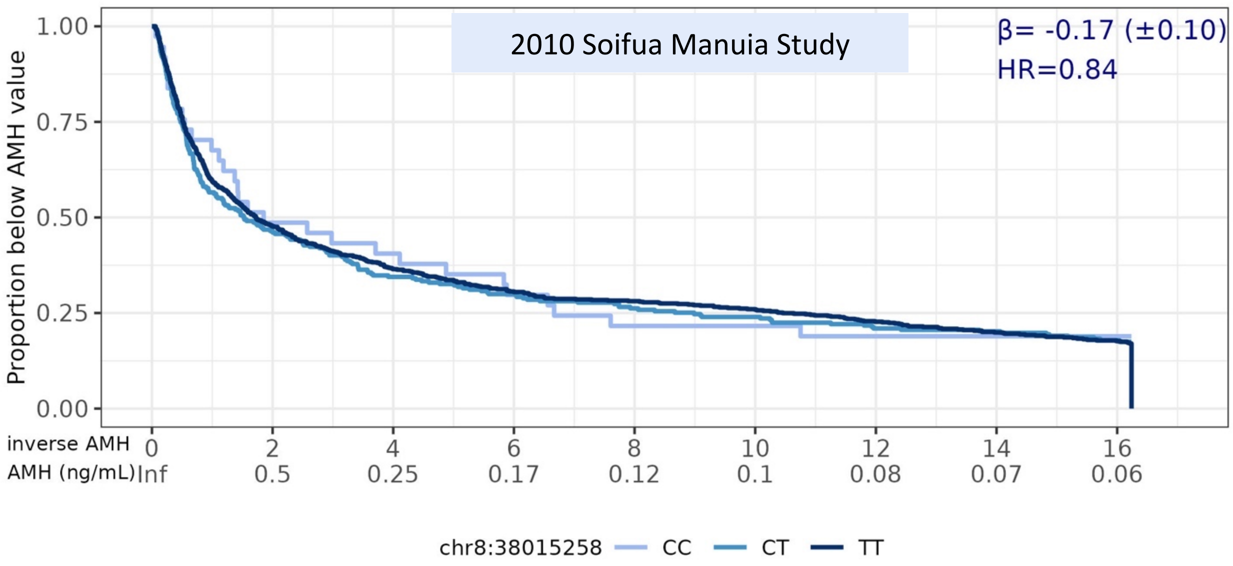

Supplementary Figure 8. AMH levels by genotype strata for 2002–03 Family Study (top) and 2010 Soifua Manuia Study (bottom) using survival curve

The inverse (or reciprocal) of the AMH level is shown in the *x* axis with the corresponding AMH levels in ng/mL beneath.

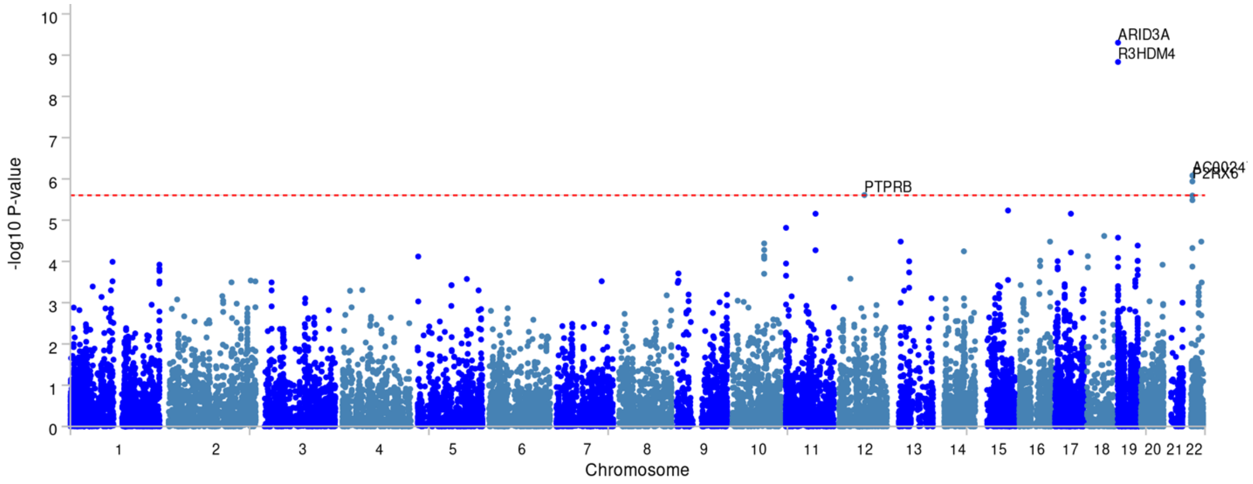

Supplementary Figure 9. Manhattan plot of the gene-based test of meta-analysis summary statistics via MAGMA implemented in FUMA

Supplementary Table 2 GWAS results with p values < 1×10^{-5}

Locus Information							2002–03 Family Study			2010 Soifua Manuia Study			Meta-Analysis
Variant (hg38)	Nearest Gene	Type	RDB	EA	EAF	EUR EAF	β (SE)	p	R^2	β (SE)	p	R^2	p
Lead SNVs from GWAS in 2002–03 Family Study													
1-230483351-A-G	*PGBD5 COG2*	intergenic	6	A	0.945	0.940	−1.12 (0.24)	1.79×10^{-6}	0.99	−0.1 (0.19)	6.05×10^{-1}	0.98	1.28×10^{-2}
5-163318201-G-C	*CCNG1*	intergenic	6	C	0.751	0.463	−0.73 (0.16)	3.70×10^{-6}	0.95	−0.02 (0.08)	7.84×10^{-1}	0.96	2.74×10^{-2}
6-16119809-G-C	*MYLIP*	intergenic	7	C	0.132	0.079	0.9 (0.19)	1.56×10^{-6}	0.99	0.09 (0.11)	4.48×10^{-1}	0.99	6.53×10^{-3}
6-123454047-G-A	*TRDN*	intronic	7	A	0.056	0.301	1.28 (0.29)	6.31×10^{-6}	0.99	0.04 (0.17)	7.98×10^{-1}	1.00	3.22×10^{-2}
6-152961137-TG-T	*FBXO5*	intergenic		-	0.050	0.000	1.33 (0.28)	1.15×10^{-6}	0.99	0.18 (0.19)	3.42×10^{-1}	1.00	3.52×10^{-3}
7-117114634-G-A	*ST7*	intronic	5	A	0.091	0.266	1.01 (0.23)	7.92×10^{-6}	0.96	0.05 (0.12)	7.03×10^{-1}	GT	2.54×10^{-2}
8-5100412-C-G	*CSMD1*	intergenic	7	C	0.860	0.854	−1.04 (0.19)	4.10×10^{-8}	0.99	0.13 (0.1)	1.97×10^{-1}	1.00	2.49×10^{-1}
8-132702524-G-A	*TMEM71*	intergenic	6	A	0.585	0.345	−0.57 (0.13)	6.62×10^{-6}	1.00	−0.03 (0.08)	7.30×10^{-1}	1.00	2.65×10^{-2}
9-87401964-G-A	*DAPK1*	intergenic	5	A	0.064	0.226	1.28 (0.28)	2.06×10^{-6}	0.99	0.02 (0.14)	8.65×10^{-1}	1.00	3.06×10^{-2}
10-117766903-A-G	*EMX2 CASC2*	intergenic	7	A	0.828	0.860	−0.79 (0.18)	6.29×10^{-6}	0.98	0.02 (0.1)	8.77×10^{-1}	0.99	7.67×10^{-2}
11-12186846-A-G	*MICAL2*	intronic	7	A	0.749	0.796	0.70 (0.16)	8.16×10^{-6}	GT	0.06 (0.09)	4.87×10^{-1}	0.98	1.18×10^{-2}
11-67922682-C-T	*UNC93B1 ALDH3B1*	intergenic	6	T	0.052	0.000	1.19 (0.28)	8.43×10^{-6}	0.99	0.28 (0.16)	9.36×10^{-2}	0.95	6.66×10^{-4}
11-74857380-CA-C	*XRRA1*	intronic		-	0.939	0.853	−1.24 (0.28)	7.58×10^{-6}	0.92	−0.02 (0.17)	9.15×10^{-1}	0.94	4.66×10^{-2}
11-131774039-G-T	*NTM*	intronic	6	T	0.507	0.502	−0.65 (0.13)	3.90×10^{-7}	0.97	−0.05 (0.08)	4.73×10^{-1}	1.00	5.16×10^{-3}
13-48149322-A-G	*MED4 RB1*	intergenic	6	A	0.925	0.955	−1.10 (0.26)	9.61×10^{-6}	0.99	−0.02 (0.14)	8.96×10^{-1}	0.99	4.65×10^{-2}
13-62687736-G-C	*LINC00448*	ncRNA intronic	6	C	0.314	0.298	−0.69 (0.14)	8.60×10^{-7}	0.99	−0.08 (0.08)	3.05×10^{-1}	1.00	2.60×10^{-3}
14-77338499-C-T	*TMED8*	down-stream	5	T	0.948	0.819	−1.71 (0.29)	3.65×10^{-10}	0.98	0 (0.18)	1.00	0.99	8.01×10^{-3}
15-73633622-A-G	*NPTN*	upstream	4	A	0.948	1.000	−1.32 (0.29)	1.83×10^{-6}	0.92	0.12 (0.19)	5.35×10^{-1}	0.95	1.45×10^{-1}
16-50822152-C-T	*CYLD*	intergenic	7	T	0.207	0.828	−0.79 (0.18)	7.75×10^{-6}	1.00	0.05 (0.09)	5.59×10^{-1}	0.99	1.73×10^{-1}
16-57904931-C-CT	*CNGB1*	intronic		T	0.085	0.003	1.11 (0.23)	1.11×10^{-6}	0.94	0.23 (0.15)	1.24×10^{-1}	0.93	5.55×10^{-4}
19-6601983-C-T	*CD70*	intergenic	5	T	0.756	0.699	0.69 (0.15)	1.97×10^{-6}	0.98	0.03 (0.09)	7.03×10^{-1}	0.98	1.84×10^{-2}
X-2162591-A-G	*DHRSX*	intergenic		A	0.941	0.994	−1.28 (0.29)	5.96×10^{-6}	0.79	−0.02 (0.17)	9.14×10^{-1}	0.76	4.41×10^{-2}
X-151747651-C-T	*CNGA2*	down-stream		T	0.374	0.343	−0.64 (0.14)	7.50×10^{-6}	GT	−0.1 (0.08)	1.91×10^{-1}	1.00	2.07×10^{-3}

Locus Information							2002–03 Family Study			2010 Soifua Manuia Study			Meta-Analysis
Lead SNVs from GWAS in 2010 Soifua Manuia Study													
Variant (hg38)	Nearest Gene	Type	RDB	EA	EAF	EUR EAF	β (SE)	*p*	R^2	β (SE)	*p*	R^2	*p*
2-36088948-A-G	*CRIM1*	intergenic	5	A	0.974	*				1.18 (0.27)	7.15×10^{-6}	0.99	
2-174701241-G-A	*(GPR155) WIPF1*	intergenic	7	A	0.807	0.913	0.00 (0.17)	9.89×10^{-1}	0.98	0.45 (0.09)	1.93×10^{-6}	0.99	1.56×10^{-5}
2-241065154-C-T	*SNED1*	intronic	5	T	0.288	0.003	−0.10 (0.15)	5.30×10^{-1}	0.99	−0.38 (0.08)	7.13×10^{-6}	0.99	1.46×10^{-5}
3-19972577-A-G	*RAB5A*	intronic	5	A	0.838	*	0.09 (0.16)	5.86×10^{-1}	0.98	−0.49 (0.1)	1.05×10^{-6}	0.98	2.74×10^{-5}
3-190080320-A-G	*TP63 P3H2*	intronic	7	A	0.910	0.804	−0.27 (0.21)	1.90×10^{-1}	0.99	0.62 (0.14)	7.84×10^{-6}	GT	4.73×10^{-4}
4-18803704-C-CA	*LCORL SLIT2*	intergenic		CA	0.477	0.713	−0.10 (0.13)	4.18×10^{-1}	0.99	0.35 (0.08)	3.27×10^{-6}	0.99	1.07×10^{-4}
4-41798742-G-A	*LIMCH1 PHOX2B TMEM33*	intergenic	7	A	0.667	0.599	−0.03 (0.14)	8.35×10^{-1}	GT	0.37 (0.08)	6.20×10^{-6}	0.89	6.15×10^{-5}
4-188298237-A-G	*TRIML1*	intergenic	5	A	0.972	0.812				1.10 (0.24)	4.64×10^{-6}	1.00	
6-5667273-C-A	*FARS2*	intronic	7	A	0.990	0.963				−1.74 (0.38)	2.27×10^{-6}	0.92	
6-58229055-C-G	*(PRIM2) NONE*	intergenic	7	C	0.967	0.732				0.97 (0.22)	8.84×10^{-6}	0.99	
8-14521938-T-C	*SGCZ*	intronic	7	T	0.969	*				−1.01 (0.22)	2.41×10^{-6}	0.98	
9-101892530-C-T	*GRIN3A*	intergenic	7	T	0.024	*				1.13 (0.23)	7.29×10^{-7}	0.99	
9-107597705-C-A	*KLF4*	intergenic	5	A	0.037	*				0.89 (0.19)	4.30×10^{-6}	0.97	
13-38915488-C-T	*FREM2*	intergenic	4	T	0.044	0.022				0.90 (0.18)	6.66×10^{-7}	1.00	
13-72599097-A-ATG	*SNORA9 MZT1*	intergenic		A	0.634	1.000	0.20 (0.14)	1.47×10^{-1}	0.96	−0.36 (0.08)	8.41×10^{-6}	0.97	6.19×10^{-4}
13-105438871-GAA-G	*DAOA-AS1*	intergenic		G	0.930	0.672	0.24 (0.27)	3.64×10^{-1}	0.95	−0.68 (0.14)	5.75×10^{-7}	0.98	3.38×10^{-5}
14-75199954-T-C	*TMED10 FOS*	intergenic	7	T	0.864	0.454	0.04 (0.19)	8.32×10^{-1}	0.90	−0.49 (0.11)	8.01×10^{-6}	0.98	7.61×10^{-5}
15-55158410-C-T	*RSL24D1*	intergenic	6	T	0.293	0.024	0.21 (0.14)	1.40×10^{-1}	0.98	−0.37 (0.08)	3.58×10^{-6}	1.00	3.50×10^{-4}
15-97854929-G-A	*LINC00923*	ncRNA intronic	7	A	0.063	0.000	0.14 (0.29)	6.31×10^{-1}	0.95	−0.72 (0.15)	1.69×10^{-6}	0.96	3.54×10^{-5}
16-85356782-G-A	*CIBAR2 GSE1*	intergenic	7	A	0.102	0.458	0.11 (0.2)	5.88×10^{-1}	0.99	0.60 (0.13)	2.92×10^{-6}	0.98	7.95×10^{-6}
18-77113272-G-A	*MBP*	intronic	7	A	0.175	0.419	−0.06 (0.18)	7.43×10^{-1}	0.99	0.41 (0.09)	8.44×10^{-6}	0.99	9.72×10^{-5}

Each SNV is presented with its gnomAD ID. The effect allele (**EA**) and its frequency in Samoans (**EAF**) followed by its frequency in Europeans in the 1000 Genome (**EUR EAF**) are reported. The effect estimates (**β**) and standard errors (**SE**) are presented except where variant MAFs were below required thresholds. **RBD** represents a score for SNV's functionality from RegulomeDB. The imputation quality score (**R^2**) is provided for imputed SNVs, where **GT** refers to genotyped variants

Supplementary Table 3 Results from transcriptome-wide analysis

Gene	Chr	Top Tissue	p	z score			
				Mean	SD	Min	Max
GINS2	**16**	**hypothalamus**	$\mathbf{2.20 \times 10^{-18}}$	**−0.372**	**1.797**	**−1.926**	**3.125**
SENP3	**17**	**pituitary**	$\mathbf{2.24 \times 10^{-7}}$	**0.382**	**2.227**	**−2.978**	**2.344**
USP7	**16**	**subcutaneous adipose**	$\mathbf{3.63 \times 10^{-7}}$	**0.657**	**2.168**	**−1.805**	**2.282**
TUSC3	**8**	**liver**	$\mathbf{4.38 \times 10^{-7}}$	**−0.206**	**1.287**	**−1.661**	**2.052**
MAFA	**8**	**subcutaneous adipose**	$\mathbf{9.30 \times 10^{-7}}$	**−0.097**	**1.425**	**−2.141**	**1.080**
METTL4	**18**	**adrenal gland**	$\mathbf{9.31 \times 10^{-7}}$	**−0.022**	**1.355**	**−2.861**	**1.191**
NDFIP1	**5**	**pancreas**	$\mathbf{2.34 \times 10^{-6}}$	**0.015**	**1.798**	**−3.650**	**2.175**
ACTR2	2	ovary	6.20×10^{-6}	−0.884	1.437	−1.753	2.096
RP11-434E6.4	16	pancreas	6.98×10^{-6}	1.151	1.496	−2.049	2.875
CHMP1A	16	liver	1.17×10^{-5}	−1.674	1.357	−3.978	−0.345
OSMR	5	liver	1.26×10^{-5}	−0.417	1.618	−2.863	2.632
ARID3A	19	pancreas	1.32×10^{-5}	3.003	1.168	1.596	4.494
MRPL44	2	ovary	1.44×10^{-5}	0.850	1.246	−0.456	3.514
PAK4	19	pancreas	2.17×10^{-5}	−0.076	1.056	−1.590	0.931
RP11-329B9.4	3	hypothalamus	2.26×10^{-5}	−0.543	1.311	−2.541	0.806
L3MBTL1	20	thyroid	2.29×10^{-5}	−0.386	1.841	−2.855	2.368
HMGCS2	1	pituitary	2.46×10^{-5}	−0.494	1.106	−2.050	1.028
RP11-329B9.5	3	pituitary	2.72×10^{-5}	−0.170	1.399	−1.920	1.392
ZNF362	1	subcutaneous adipose	3.07×10^{-5}	0.017	1.369	−2.075	1.193
MLH3	14	adrenal gland	3.60×10^{-5}	3.481	1.005	1.292	4.005
ABCD4	14	hypothalamus	4.12×10^{-5}	1.880	1.709	−1.074	3.568
LYZ	12	liver	4.23×10^{-5}	0.179	1.197	−1.004	1.776
RPP25	15	subcutaneous adipose	4.63×10^{-5}	−0.503	1.023	−1.250	0.637
RMI2	16	ovary	4.89×10^{-5}	1.925	1.524	−1.037	2.948

Statistically significant findings are represented by p values depicted in bold.

Supplementary Table 4 Look-up of known AMH loci in Samoan GWAS.

AMH loci from previous GWASs of European ancestry								Samoan GWAS				Most significant variant within ±50 kb of the AMH loci identified in previous GWASs				Samoan		EUR		Samoan GWAS		
rsID	gnomAD ID (hg38)	Nearest Gene	EA	EUR EAF	β (SE)	*p* value	Study	R^2	Samoan EAF	Effect Direction	*P*	gnomAD ID (hg38)	Functional Conse-quence	Distance (bp)	EA	EAF	LD (r^2)	EAF gnomAD	LD (r^2)	Effect Direction	*P*	Bonferroni corrected threshold
rs6729614	2-144887307-A-G	*TEX41*	G	0.26	0.08 (0.01)	5.56×10^{-11}	[1]	1.00	0.012	Not examined		2-144839456-A-T	ncRNA intronic	−47,851	A	0.81	0.015	0.3783	0.0874	−−	0.0018	0.0028
rs11683493	2-173394597-C-T	*CDCA7*	T	0.57	−0.08 (0.01)	1.68×10^{-8}	[2]	1.00	0.349	−+	0.573											
rs116090962	5-146560687-G-A	*CTB-99A3.1*	A	0.02	0.38 (0.07)	6.00×10^{-9}	[2]	0.99	0.0003	Not examined		no variant with $p < 0.05$ within ±50 kb of rs116090962										
**rs10093345**	**8-38015258-C-T**	***EIF4EBP1***	**T**	**0.72**	**−0.08 (0.01)**	$\mathbf{5.05 \times 10^{-9}}$	[1]	**0.97**	**0.825**	**−−**	**0.016**											
rs762643	14-53956049-G-T	*BMP4*	T	0.44	−0.07 (0.01)	3.99×10^{-9}	[1]	1.00	0.338	+−	0.499											
rs10417628	19-2251818-T-C	*AMH*	C	0.97	0.32 (0.04)	9.56×10^{-12}	[1]	0.09	0.996	Not examined		19-2250470-G-A	intronic	−1,348	A	0.47	0.004	0.0419	0.0009	++	0.0006	0.0011
rs16991615	20-5967581-G-A	*MCM8*	A	0.06	0.16 (0.02)	4.68×10^{-9}	[1]	1.00	0.0007	Not examined		20-5993582-A-G	p.R773G	26,001	A	0.83	0.001	0.00001	*	++	0.0299	0.0026
rs186430430	22-28707610-T-C	*CHEK2*	C	0.002	0.79 (0.12)	9.69×10^{-11}	[1]	0.08	0.00002	Not examined		22-28703766-T-C	intronic	−3,844	T	0.84	NA	0.4825	0.0037	−−	0.0051	0.0019

The variant info for the lead SNVs identified in AMH GWASs from women of European ancestry (**EUR**) is under the light blue header. For the lead variant in each known AMH locus, we report the **rsID**, **gnomAD ID**, **nearest gene**, effect allele (**EA**), effect allele frequency (**EAF**) in the originating study, effect size and standard error (**β (SE)**), ***p* value**, and reference to the originating study: [1)] (Pujol-Gualdo *et al.*, 2024) [2)] (Verdiesen *et al.*, 2022)
Next, under the center left dark blue header, we provide imputation quality (***R*²**), **EAF** in Samoans, **effect directions** in the meta-analyzed samples, and the ***p* value** from the **Samoan GWAS** for the same SNV, if the allele frequency of the SNV was high enough to be included in the meta-analysis (see Methods).
The next seven columns contain information about the lead variant in the Samoan GWAS within ±50 kb of the lead variant from prior European GWASs if the lead variant was not examined in the Samoan GWAS and if the nearby variant had $p < 0.05$. For each SNV, we provide the **gnomAD ID**, **functional consequence**, **distance** from the prior GWAS lead SNV, the **EAF** and the **LD *r*²** with the prior GWAS lead SNV in Samoans, and the **EAF** (gnomAD v4) and **LD *r*²** with the prior GWAS lead SNV in Europeans (1000G EUR, calculated using LDpair function in LDlink from NIH). An asterisk (*****) indicates that the variant was not present in 1000 Genomes.

The final three columns, under the righthand dark blue header, contain the **effect directions** in the meta-analyzed samples, the ***p* value** from the Samoan GWAS, and the per-region **Bonferroni-corrected threshold** for statistical significance.
The AMH loci from previous GWASs that replicated in Samoan meta-analysis are in bold.
